# A new emphasis on root traits for perennial grass and legume varieties with environmental and ecological benefits

**DOI:** 10.1002/fes3.78

**Published:** 2016-01-28

**Authors:** Athole H. Marshall, Rosemary P. Collins, Mike W. Humphreys, John Scullion

**Affiliations:** ^1^ Institute of Biological and Environmental ResearchAberystwyth UniversityGogerddanAberystwythSY233EEUK

**Keywords:** Ecology, environment, grasslands, phenotyping, plant breeding, roots

## Abstract

Grasslands cover a significant proportion of the agricultural land within the UK and across the EU, providing a relatively cheap source of feed for ruminants and supporting the production of meat, wool and milk from grazing animals. Delivering efficient animal production from grassland systems has traditionally been the primary focus of grassland‐based research. But there is increasing recognition of the ecological and environmental benefits of these grassland systems and the importance of the interaction between their component plants and a host of other biological organisms in the soil and in adjoining habitats. Many of the ecological and environmental benefits provided by grasslands emanate from the interactions between the roots of plant species and the soil in which they grow. We review current knowledge on the role of grassland ecosystems in delivering ecological and environmental benefits. We will consider how improved grassland can deliver these benefits, and the potential opportunities for plant breeding to improve specific traits that will enhance these benefits whilst maintaining forage production for livestock consumption. Opportunities for exploiting new plant breeding approaches, including high throughput phenotyping, and for introducing traits from closely related species are discussed.

## Introduction

Grasslands are the main survival resource for about 1 billion people worldwide (Peyraud et al. 2014) and cover nearly 70% of the world's agricultural area (Sousanna and Luscher 2007). They are defined as ‘land devoted to the production of forage for harvest by grazing/browsing, cutting or both, or used for other agricultural purposes such as renewable energy production’ (Peeters et al. 2014). In Europe (EU‐27), grassland accounts for 39% of the agricultural area (Huyghe et al. 2014). The main reason for the prevalence of grasslands in agriculture worldwide is that they provide a relatively cheap source of feed for ruminants and allow the production of meat, wool, and milk from grazing animals. Furthermore, they are located frequently in marginal land areas deemed otherwise unsuitable for most other agricultural crops. Aside from those natural grasslands considered to be climatically determined, it is grasslands that are anthropogenically generated that have the most significant role in agriculture and are the focus of this review. They are located mainly within temperate climatic regions, where woody vegetation is excluded and herbaceous plant communities are maintained by appropriate human intervention and by livestock agriculture. It is possible to further divide these anthropogenically derived grasslands into long‐term naturalized grasslands and those that are cultivated, which differ according to level of intensification. The latter, often termed ‘improved grasslands’, vary markedly in their coverage across the four countries of the UK, occupying 13.8% of Scotland, 30.3% of England, 42.0% of Wales, and 54.0% of Northern Ireland according to LCM2000 calibrations (Fuller et al. 2002). High levels of grassland improvement in Northern Ireland reflect the importance of livestock farming to the country's economy in comparison to the rest of UK (Cruickshank 1987), whereas economic pressures associated with a smaller average farm size compared to those in England and Scotland are believed to have contributed to the prevalence of the practice in Wales (Eadie 1984).

The capacity of improved grasslands to sustain animal production has been their primary function and consequently the main focus of research for most of the last century. Intensive grassland systems in the UK are currently associated with the widespread use of monocultures (usually perennial ryegrass *Lolium perenne* L.) or binary mixtures that include a legume (usually white clover *Trifolium repens* L.). These swards have a high yield potential and feeding value, can sustain frequent harvesting and/or high stocking rates, and are maintained by moderate‐to‐high levels of nitrogen (N) input (Wilkins et al. 2002). Peukert et al. (2014) found that rates of soil and phosphorus (P), but not N, losses under intensively managed grassland could be as high as those for other agricultural systems. Such intensive grassland systems are often short‐lived and temporary components within a managed crop‐rotation and as such have only a limited environmental benefit.

More extensive grassland designs and management practices require the use of mixtures of complementary perennial species in order to achieve more sustainable crop production and greater crop persistency for five‐ten years or more before the need for re‐sowing. Such “long‐lived” pastures can be regarded as multifunctional, and in addition to their provision of forage for livestock use, they also provide a habitat for a vast and diverse ecosystem that can support a multitude of “hidden” attributes in terms of alternative environmental benefits. Grassland ecosystems are dependent on, and affect, a host of other biological organisms in the soil and in adjoining habitats. These organisms provide services such as decomposition, maintenance of soil fertility and provision of clean water. The grassland acts as a resource for insects, such as bees, required to ensure pollination and future development of populations of wild and cultivated plant species. Grasslands ecosystems also support large populations of invertebrates, many such as earthworms providing key food resources for birds and mammals (Kruuk and Parish 1981; Peach et al. 2004). These functions are often collectively termed “ecosystem services” (Tancoigne et al. 2014) and have wider benefits such as prevention of soil erosion, carbon sequestration, and genetic conservation. Improved soil structure, nutrient retention, and N‐fixation comprise some of the additional benefits derived from improved grasslands. These grasslands are now increasingly recognized for their wider contribution to society and their delivery of “public good” (Abberton et al. 2008; Humphreys et al. 2014a).

An important but little acknowledged feature of improved and permanent grassland is the enormous biomass perennial grassland species produce below ground (Newman et al. 1989) – a feature that is considered highly relevant to the provision of ecosystem services (Bardgett et al. 2014). A survey of old grassland in the UK carried out by Dickinson and Polwart (1982), for example, measured a root biomass of around 5 t/ha in the top 15 cm of soil. Plant breeding effort has focused primarily on above‐ground traits and has largely neglected the potential to improve root design and function, mainly due to difficulties in root phenotyping and selection (Jahufer et al. 2008). In any case, forage crops grown in highly fertilized monocultures have maximum above ground production and forage quality as the main breeding objectives; here the form and function of the root system are considered to be relatively unimportant. As a result of these factors, many of the varieties of our current forage species are relatively shallow rooting, a trait that will compromise both their long‐term persistency and yield potential following onset of stress conditions (Humphreys et al. 2014). Many of the ecological and environmental benefits provided by grasslands emanate from the interactions between the roots of different plant species and the soil in which they grow. Therefore, it is essential that modern plant breeding strategies aiming to promote sustainable crop production take into account the overall effects of growth by the crop on the surrounding biota and ecosystem.

Here, we review current knowledge on the role of grassland ecosystems in delivering ecological and environmental benefits related to soil structure and functioning. The plant breeding focus will be predominantly on root traits, and will also consider how new developments in dynamic root imaging, combined with an increased understanding of the genetic bases of variation in root architecture, could bring about a step‐change in our awareness of these root traits. This understanding will allow traits such as root ontogeny, depth, thickness, and distribution to be designed strategically to produce an optimal balance between above‐ground biomass productivity and below‐ground biotic and abiotic interactions. The review focuses primarily on UK grasslands but many of the concepts and approaches discussed are applicable to the wider temperate grassland areas and to grassland systems in other climatic zones.

## Environmental and Ecological Benefits from Grassland

### Regulation of water release and flood prevention

Grasslands, particularly in temperate regions, frequently occur in land areas where rainfall is the highest, which for the UK is predominately in western and northern regions. These grasslands are important as catchments for major rivers, where they regulate water acquisition, its quality and its later release from soils. Climate change will inevitably lead to changes in agro‐ecosystem functioning. The most recent Intergovernmental Panel on Climate Change (IPCC) report predicts major increases in global mean air temperatures of between 1.8 and 4.0°C by 2100, bringing with it greater uncertainty in weather patterns and also an increased incidence of extreme events (IPCC 2013; WMO 2013). Heavy rainfall impacts on the capacity of agricultural grasslands to effectively retain the rainwater that falls on them and to regulate its rate of loss, thereby leading to significant run‐off and flooding of lowland areas. In this regard, the study by Peukert et al. (2014) noted several rainfall events where the runoff coefficient on intensively managed grassland exceeded 100%; these findings point to the need to enhance the capacity of grassland systems to deliver effective water regulation.

The incidence of extreme weather events has become more frequent in the last 20 years (WMO 2013). For example, in Europe, flood and drought events currently estimated to have a frequency of one per 100 years are now predicted to recur every 10–50 years by the 2070s (Lehner et al. 2006; WMO 2013). In temperate European catchments, increased volumes and intensity of winter rainfall are predicted, leading to raised levels of erosive runoff (Sauerborn et al. 1999). The immediate impact of flooding on crop production alone in 2014 in England was estimated at £25 million (ADAS 2014). However, the socio‐economic after‐effects of these extreme weather events may also persist for many years after the event has occurred, and in some cases have led to destabilization of local communities (Lehner et al. 2006). Despite these risks, our understanding of how extreme events will impact on crops and soil functioning, and consequently on sustainable livestock farming, remains poor and requires urgent attention.

Hydrologists have long been aware of the pivotal role of vegetation in regulating and buffering the hydrological cycle. Changes in land use have been shown to change the regional climate (Stohlgren et al. 1998). At the single plant scale, biophysical changes in soil hydraulic properties due to root activity have also been demonstrated (Whalley et al. 2005). Thus, root activity tends to increase the number of large pores in soils, and there is a tendency for this property to change their water release characteristics. Detailed studies have illustrated the importance of rooting depth and the vertical variation in root function on soil water uptake. They have also highlighted that soil porosity is not a fixed parameter and that its dynamics are strongly influenced by vegetation (Rodriguez‐Iturbe et al. 2001). Rooting depth determines the soil volume from which plants draw water and is influenced by various key soil hydraulic properties; soil texture and rooting depth largely define the plant‐available water capacity (MacLeod et al. 2007). Recent unpublished results using X‐ray Computed Tomography have revealed very different impacts on soil porosity derived from *Lolium, Festuca, Festulolium*, and *Trifolium* roots over time and have shown how these effects changed with contrasting soil types (Mooney S.J., pers comm.).

MacLeod et al. (2013) reported how a deep rooting *Festulolium* (a hybrid between a ryegrass and fescue species) variety reduced rainfall run‐off compared to both its parental species by 51% compared to perennial ryegrass, and by 43% compared to meadow fescue (*Festuca pratensis*). The *Festulolium* variety was a synthetic version of *Festulolium loliaceum*, a natural grass hybrid involving these ryegrass and fescue species which occurs naturally in waterlogged soils in mature water meadows. Frequently in this habitat its parental species are either absent or present in only low numbers, implying that the hybrid has a selective advantage (Humphreys and Harper 2008). MacLeod et al. (2013) suggest that the deep rooting of the *Festulolium* variety and its subsequent dieback, particularly at depth in the root profile, were enhancing soil structure and through this assisting water retention. Among the temperate legumes, red clover (*Trifolium pratense*) is deeper rooting than white clover (*T. repens*) and while its effects on reducing run‐off are currently unknown, its potential benefits in this regard are currently under investigation at IBERS. These investigations follow a dual strategy to produce new grasses and legumes that (i) provide nutritious forage for livestock, and (ii) possess enhanced root traits that can improve soil hydrology, and are carried out as part of a new BBSRC‐LINK Programme, SUREROOT (www.sureroot.uk). The novel grasses and legumes are being assessed under diverse livestock management systems at different UK locations. For the first time, two new UK national capabilities facilities are being used in conjunction: IBERS’ National Plant Phenomics Centre (NPPC) in Aberystwyth and Rothamsted Research's Farm Platform at North Wyke, Devon. Detailed analysis of changes in root architecture and ontogeny are identified in the NPPC, and their impact on soil hydrology at the field scale is measured on the Farm Platform. Here, the fields are isolated hydrologically to enable rainfall run‐off to be accurately determined. The soils at North Wyke are representative of many in western England, are shallow and cover highly impermeable clay‐layers, and are thus prone to flooding. Over an 80 h period in November 2012, more than 46 × 10^6 ^L of rain fell on the North Wyke Farm Platform, of which 90% was lost as overland flow or in drainage (P. Murray, pers. comm.), illustrating the importance of improving the capacity of grassland to retain water.

Where surface runoff occurs, there is the potential for soil erosion. Grasslands have in general been considered to reduce water erosion in comparison with arable‐cropped land, although this view is being increasingly challenged, particularly in relation to impacts on aquatic ecosystems. Protection of the soil surface from raindrop impact, higher surface infiltration rates and enhanced stabilization of otherwise erodible fine soil particles into larger water‐stable aggregates are considered mechanisms by which grasslands restrict erosion. It is clear, however, that where these benefits are not achieved grasslands may fail to deliver soil conservation objectives. The physical changes that promote soil conservation arise directly from physical binding by roots, but also through a complex interplay between roots and the soil biological community, driven by root‐derived inputs of C. For example, there is abundant evidence that earthworm burrows make a major contribution to surface infiltration, reducing overland flow (e.g., Shipitalo and Butt 1999), and that grasslands support large populations of these burrowing earthworms (e.g., Scullion et al. 2002).

### Carbon (C) sequestration

Reducing the “carbon footprint” of food production is fundamental for developing globally sustainable agriculture to support current human population growth in a changing climate. Because of the large amounts of C held as organic matter (SOM) in the world's soils, it is very important to understand how best to conserve or, if possible, increase soil C stocks (Powlson et al. 2014).The cultivation of perennial crops with large and deep rooting systems to increase the input of atmospheric CO_2_ to agricultural soils has recently been highlighted as a potential approach for reducing the impact of agricultural production systems on greenhouse gas emissions (Kell 2011).

Whether or not an ecosystem (above‐ and below‐ground) accumulates or loses C is a function of input and output. Sequestration of C occurs when gross primary productivity (GPP) exceeds ecosystem respiration (ER), which in turn is the sum of plant respiration and heterotrophic respiration of nonphotosynthetic organisms. This has been termed net ecosystem productivity (NEP) (Chapin et al. 2006). The final rate of accumulation or loss of C in a particular ecosystem (net ecosystem carbon budget (NECB)), in addition to NEP, will depend on external deposition of C (such as inputs of organic manures and dissolved C in rain water) and also loss through erosion, removal (harvesting), and nonbiological oxidation through fire or UV radiation (Lovett et al. 2006).

We believe that should opportunities for C sequestration in grasslands be enhanced through the widespread use of new varieties with high root biomass turn‐over, particularly at depth, it may well provide a low‐cost short‐term measure to mitigate atmospheric C accumulation until “low” or “zero C” energy sources have opportunities to take effect. The strategy would have advantages over the high economic and environmental costs associated with long‐term measures such as engineering techniques of CO_2_ capture and injection into geological and oceanic strata.

Plant roots constitute a significant proportion of the C transferred to soils as mediated by processes in the rhizosphere, and perennial plants such as grasses offer significant advantages over annuals as a means of increasing soil C stocks because of their prolonged photosynthetic activity, greater root biomass, and proportionately deeper rooting. Furthermore, grasslands have an advantage over arable crops in not requiring annual ploughing and resowing, which through the soil disturbance generated will inadvertently cause losses of C into the environment.

Kell (2011) highlighted a key focus of research to be root–soil interactions that govern SOM dynamics and the possibility of breeding plants with root traits to deposit more or more‐recalcitrant C in soils, especially at depth. In addition to contributing to atmospheric CO_2_ sequestration, increased root C deposition may benefit soil quality and function, through better soil physical structural stability, and by favoring beneficial biological communities in the rhizosphere (Peiffer et al. 2013). However, it should be noted that plant breeding activity targeted at improved C sequestration in soils is both highly problematic and complex, and its effects are dependent on many interacting factors. Plant‐derived inputs of C to soil include root turnover, root exudation, and mycorrhizal turnover. Each of these has different seasonal and spatial dynamics in the soil, and different dependencies on plant and soil conditions. For example, a plant that maintains roots for longer (i.e., less root turnover) may allocate less C to the production of new roots, but expend more C and energy on maintaining old roots that may be less efficient in nutrient and water capture (Norby and Jackson 2000).

The capacity of a particular soil to accumulate SOM is finite and depends on a complicated interaction between physical, chemical and biological processes (Powlson et al. 2007). It is now acknowledged that new inputs of fresh plant‐derived C and other root‐induced changes in the soil may have a totally contrary effect to that desired, and may stimulate the turnover of existing SOM and nutrients by soil microbes. Reports of the effects of such “priming” record instances where fresh labile organic matter was found to stimulate the decomposition of older SOM throughout the soil profile (Kuzyakov 2010) and more specifically at depth (Fontaine et al. 2007). What happens to soil C stocks at depth during disturbances such as drying or ploughing, land‐use change, etc. is still little understood (Gregory et al. 2011).

Deeper and more highly branched rooting will increase the contact of organic matter, both root exudates and decomposing roots, with soil minerals, and may have an important benefit in terms of soil C sequestration (Jobbagy and Jackson 2000; Kell 2011, 2012). In addition, root C appears to make a higher contribution to soil C than above‐ground inputs, especially at depth (Rasse et al. 2005; Mendez‐Millan et al. 2010). Deep soil C is an important contributor to overall soil C stocks. Gregory et al. (2011) estimated that 980 Mt of organic C is stored below 30 cm depth in soils in England and Wales, approximately 50% of the total. Evidence from tropical savannah also suggests that the planting of exotic deeper rooting plant varieties leads to significant increases in soil C stocks (Fisher et al. 1994). However, in order for deep rooting plants to have a significant effect, the soil must be deeper than the normal rooting depth and root growth must not be impeded by compaction, nutrient limitation, or waterlogging. On thin soils, or where roots cannot penetrate, deeper rooting plants are not going to provide additional sequestration benefits.

Pasture improvement and the introduction of high yielding forage grasses and legumes have been shown both theoretically (e.g., Soussanna et al. 2004) and practically (e.g., Lal et al. 1998) to substantially increase C stocks as compared to that achieved from some preexisting grasslands or land under arable farming. For example, compared to annuals, the sowing of perennial crops such as *Festuca arundinacea* increased soil C stocks by 17.2% (equivalent to C sequestration of circa 3 Mg C/ha/year over a 6‐year period). As far as we are aware, no attempts have been made to breed explicitly for increased soil C sequestration in any economically important temperate forage species, mainly due to unanswered questions regarding (a) the magnitude, (b) genetic control, and (c) G × E modulation of the various C flux pathways between plant, soil and atmosphere. Consequently, uncertainties about which traits are most likely to confer durable increases in C sequestration remain. The feasibility of evaluating traits likely to be linked with C sequestration and subsequently identifying the associated QTLs (thereby enabling marker‐assisted selection) is currently under investigation at IBERS.

### Improvement of soil structure

Grassland farming relies largely on long‐term production from perennial species, and sustainable grassland production consequently depends on the maintenance of good soil quality. Inherent soil characteristics (e.g., texture and mineralogy) form a significant part of “soil quality”, and these are not readily amenable to human manipulation. However, other aspects of soil quality are more dynamic in nature, being strongly affected by recent land use. Such attributes include SOM (discussed in Section ‘Carbon (C) sequestration’) and soil structural properties (e.g., porosity, permeability, and aggregation) (Carter 2000).

There is an increasing body of evidence that plants differ in their effects on soil structure (Materechera et al. 1994; MacLeod et al. 2007). Soil structure is commonly interpreted through the concept of an aggregate hierarchy (Tisdall and Oades 1982; Miller and Jastrow 1996), and comparative studies have shown that differences in aggregating and stabilizing efficiency in soils vary not only between plant species (Drury et al. 1991; Materechera et al. 1994), but also between varieties within species (Carter et al. 1994), and even potentially between genotypes within varieties (MacLeod et al. 2007). These effects may be direct, resulting from the influence of morphological root traits on binding soil particles, or indirect, for example, through associated biological action on aggregate formation. The latter effects may have a stronger impact on soil structure (Bardgett et al. 2014).

#### Direct effects

Several investigations have been carried out on forage plant‐driven changes in soil structure, and some have compared the effects of different plant species. Mytton et al. (1993) and Holtham et al. (2007) both described visible differences between white clover and perennial ryegrass soil cores in terms of particle aggregation, with much greater aggregation present in the white clover cores. In the latter study, while the soil in the white clover cores was more structured than that in the perennial ryegrass cores, the root biomass present in white clover was substantially lower. Aggregation of soil particles near white clover roots was also observed in glass‐fronted rhizotrons by Pugh et al. (1995). The process of aggregation is important for many aspects of soil functioning related to plant growth, and soils with more stable aggregates are also more resistant to surface crusting (Le Bissonnais and Arrouyais 1997), and to compaction (Angers et al. 1987). Such soils consequently favor seedling emergence, root growth, and water infiltration and storage (Angers and Caron 1998). Increases in aggregation also contribute to SOM build‐up and thus to nutrient storage. In another component of the study of Mytton et al. (1993), significant improvements in drainage in soil cores containing white clover were observed, founded on differences in soil macropore space. Values of soil macroporosity for pure perennial ryegrass and white clover soil cores were 23.6% and 45.3%, respectively. Holtham et al. (2007) also recorded substantially higher rates of drainage from soil cores under white clover (599 ml/day) than under perennial ryegrass (115 mL/day). They attributed this large difference to the effects of local structuring around the clover roots, creating a more porous soil. The pore structure of the cores was simulated using a void space network model (“Pore‐Cor”). This confirmed larger pores beneath white clover, a difference between the species in local soil structuring and a saturated hydraulic conductivity (describing the ease with which water can move through pore spaces) in white clover that was four times greater than under perennial ryegrass. The formation of continuous macropores, key for promoting conductivity, by penetrating roots represents a significant plant‐induced change in soil structure. Macropores facilitate aeration, and water movement and temporary storage in the soil, as well as decreasing resistance to further root growth (Angers and Caron 1998). Soil macroporosity and pore size distribution were also measured by Papadopoulos et al. (2006), using high‐resolution image analysis in a study comparing soil structuring under five plant‐type treatments in a stockless organic rotation. The only inputs to the plots were plant biomass from the treatments themselves. In the spring of the second year of the rotations, the two red clover/perennial ryegrass treatments had by far the highest values of macroporosity and the vetch treatment had the lowest. The high soil porosity values initially observed by Papadopoulos et al. (2006) in the red clover treatments were attributed to the way in which this species rapidly develops a high‐ density root mat. However, differences between treatments were transient, and by later in the summer had disappeared. Three years later, when all plots had grown winter wheat, it was found that macroporosity and pore size were similar in all plots. Thus, it appears that the measurable and rapid improvements in soil structure and aggregate stability brought about by legume species may not be robust and could be quickly reversed by subsequent arable crops in a rotation system. In longer term grassland systems, however, the positive effects of legumes may persist.

The roots of growing plants help to aerate the soil by creating channels through which water, soil solutions, microorganisms, and soil invertebrates can move easily. Root system architecture and depth distribution are consequently important contributors to soil quality and therefore merit attention. Broad interspecific differences have been identified between perennial ryegrass and white clover in terms of root dimensions (e.g., mean root diameters of 0.19 and 0.26 mm, respectively, measured by Evans (1977)). Significant intraspecific differences in root system morphology between white clover populations grown as spaced plants in the field have also been described, with some varieties producing large tap‐rooted systems with a small proportion of finer, fibrous roots, and others producing no large tap‐roots and a high proportion of fibrous roots (Caradus 1977). However, more detailed information on root architecture (e.g., the proportions of root lengths produced in different diameter size classes at different depths in the root profile) is lacking, due to the technical difficulties associated with measuring plant roots *in situ*.

#### Indirect effects

Soil aggregation processes result from the production of organic‐binding agents (e.g., polysaccharides) by microbes, from microorganisms breaking down organic matter, and through the enmeshing effects of plant roots and fungal hyphae (Watson et al. 2002). Due to the temporary nature of plant roots and fungal hyphae (with tissue turnover), their ability to act as soil structuring agents is related to their rates of production and longevity, along with the effects of root products on the activity of the soil biotic community. This suggests that the importance of root system architecture to the formation and stabilization of soil aggregates is related to the kinds of roots produced, as well as to other factors associated with the mycorrhizal condition of the roots (Miller and Jastrow 1990). Specifically, it is thought that coarser‐rooted plants are more likely to be dependent on a mycorrhizal association than finer, fibrous‐rooted plants (Heterick et al. 1992), and are therefore more likely to have a greater affinity for extra‐radicle fungal hyphae in the soil (Miller and Jastrow 1996). Perennial legume species tend to have a high dependence on associations with mycorrhizal fungi, except under high‐P conditions (Heterick et al. 1992). It has therefore been proposed that a major part of the influence of legumes on soil structure results from the interaction of their root systems with associated mycorrhizas (Miller and Jastrow 1996). In contrast, perennial ryegrass usually shows no, or only a small positive response to inoculation with mycorrhizas in terms of plant growth and nutrient uptake (Hall et al. 1984). This has been attributed to the fact that perennial ryegrass has an extensive, finely divided root system (Evans 1977), and therefore benefits less from the mycorrhizal symbiosis. In addition to the potential benefits for soil structure arising from physical binding by fungal hyphae, their input of glomalin‐like compounds (Rillig and Mummey 2006) has also been implicated in the stabilization of soil aggregates. There is again evidence that mycorrhizas vary in the extent of these inputs and that this variation is affected by root‐fungal interrelationships.

### Farmland biodiversity

There has been a general deterioration in biodiversity on UK farmland over recent decades with declines seen in plant, insect, and bird populations (Firbank et al. 2013). Grasslands have the potential to deliver biodiversity services but they vary in the extent and nature of the benefits that they deliver. In some aspects (e.g., pollinating insects), the benefits relate to the diversity and management of the plant communities present. Productive grasslands tend to have a limited range of plant species present and any biodiversity benefits relate mainly to their support of large soil faunal communities (e.g., Scullion et al. 2002) based on limited disturbance and large inputs of organic residues; these communities provide a substantial input to above‐ground food chains benefitting a range of farmland birds and mammals. Alterations to rooting traits, and associated C inputs to soils, have the potential to affect both soil and above‐ground communities. Although there are a number of soil invertebrates contributing to this link, earthworms have been studied most intensively.

A number of the farmland bird species currently in decline forage extensively on grasslands for earthworms and other soil invertebrates. For example, Peach et al. (2004) concluded that recovery of song thrush (*Turdus philomelos*) populations in lowland Britain will require an increase in grassland cover to support these food resources. In addition, maintenance of moist soil surfaces during early summer is necessary to increase prey availability and improve recruitment of fledglings into breeding populations.

Farmland mammals show a similar reliance on prey that are present in greater abundance in grassland compared with other agricultural land uses. Badgers (*Meles meles*) have a strong seasonal dependence on earthworms and insect larvae in their diet (Cleary et al. 2011). These species and others such as moles (*Talpa europaea*) and foxes (*Vulpes vulpes*), have a preference for the earthworm species *Lumbricus terrestris* in their diet, indicating that the composition of prey communities may be as important as their overall biomass (Murchie and Gordon 2013).

Grass breeding initiatives that enhance C input to soils via their root systems are likely to increase grassland soil faunal populations overall. Where changes to root architecture favor a redistribution of C inputs to greater depths, this change may affect the composition of these populations, for example, promoting deeper burrowing earthworm species such as *L. terrestris,* rather than those inhabiting surface layers. Impacts of changes in root architecture on soil profile moisture regimes may also affect prey availability. Greater storage of incident rainfall (see Section ‘Regulation of water release and flood prevention’) and enhanced water uptake from depth may prolong the period over which surface soils are moist enough to facilitate near surface feeding on soil invertebrates. Therefore, future plant breeding programs may have much wider impacts on agroecosystems than those focussed on soil characteristics alone.

## Implications for Plant Breeding Programs

### Selection for above versus below ground biomass in forage species

Until recently, relatively little emphasis has been placed in forage species on selection for below‐ground biomass, despite recognition of the fact that root system morphology (size and architecture) is crucially important not only for provision of the long‐term ecosystem services described above, but also for the basic functions of water and nutrient uptake. Genetic improvement of forage grasses and legumes has traditionally focused on the selection of traits that improve sward yield and animal performance, for example, by increasing production of above‐ground biomass and improving forage quality characteristics and plant persistence. The majority of these traits are included in the statutory evaluation of varieties in official trials. However, the major challenges facing forage plant breeders now include selection for traits that deliver environmental benefit (e.g., larger, deeper root systems), and dealing with any resultant trade‐offs between environmental and production traits. It is well known that improvements in above‐ground yield traits in forage species do not necessarily result in larger root systems, and this may impair the capacity of new varieties to deliver efficient water and nutrient uptake combined with environmental benefits (Crush et al. 2010). The presence of appropriate root system traits in forage species has measurable effects on plant performance under conditions of drought and nutrient stress. In white clover, for example, differences in root system depth were shown to be strongly related to the performance of cultivars and ecotypes under drought stress (Ennos 1985). Also in this species, root traits have been shown to affect the uptake of nutrients such as P (Nichols et al. 2014a,b). In this case, root length and frequency of branching were identified as the key traits for P uptake.

Focusing on white clover, the most widely grown temperate forage legume, some research has been carried out to quantify the potential for genetic improvement of root traits (Ennos 1985; Caradus and Woodfield 1990, 1998; Woodfield and Caradus 1990; Nichols et al. 2007; Jahufer et al. 2008). Significant genetic variation has been observed, and it is thought that a major component of variation in root growth is due to additive gene effects which can be selected for (Woodfield and Caradus 1990). Reported heritability for root system dry weight was higher than for shoot dry weight, and heritabilities for taproot diameter and proportion of the roots with a diameter greater than 1 mm were both high enough to make these traits amenable to selection (Caradus and Woodfield 1990). However, the presence of strong correlations between shoot and root traits within white clover morpho‐types (e.g., leaf‐size categories) presents a complication when selecting for root characters in this species (Annichiarico et al. 2015). Indirect selection for root traits to enhance drought tolerance has been carried out in white clover, and the results showed limited scope for improvement within the morphotype of germplasm used (Annicchiarico and Piano 2004). The absence of an effect of root traits (in this case overall root biomass) on drought tolerance in the latter study was considered to be due to the existence of concurrent, less efficient methods for controlling water loss. Thus, it appears that the strong correlations in white clover between the shoot and root system morphology may result in a conflict, when considering selection for drought tolerance, between the improved water uptake associated with the deeper and more extensive root systems of larger‐leaved, more tap‐rooted morphotypes and the superior water conservation associated with small leaf size (Woodfield and Caradus 1987). An alternative approach to improving drought tolerance in white clover involves its hybridization with other, closely related species that exhibit this trait (e.g., Marshall et al. 2001; Nichols et al. 2014a,b). The introgression into white clover of the rhizomatous growth habit from Caucasian clover, *Trifolium ambiguum* (Marshall et al. 2001) has shown promise for improving drought tolerance (Marshall et al. 2015). The latter study concluded that the greater biomass at depth in the root profile in backcross hybrids of the two species contributed to their superior drought tolerance compared with white clover. This change in root system shape was not achieved at the expense of reductions in forage yield or quality in the hybrid germplasm (Marshall et al. 2003, 2004). Nichols et al. (2014a,b) showed that above‐ground yield in first generation backcross hybrids between white clover and *Trifolium uniflorum* was significantly less affected by long‐term drought than the white clover parent, and that biomass allocation to roots increased.

In forage grasses, alternatives to ryegrasses, for example, tall fescue, with large deep root systems and more efficient water use have been used regularly in areas such as in the USA where water supply is suboptimal and droughts occur regularly. *Festulolium*‐based research and breeding is gaining increasing acceptance as a way forward that can harness the attributes of rapid growth rates, establishment, and forage quality traits present in ryegrasses with the enhanced stress resistance, root depth, and strength traits found in the fescues (Ghesquiére et al. 2010). The variety “Lueur” developed in France, an amphiploid hybrid combination of *L. multiflorum* and *F. glaucescens*, has been shown to extract water more effectively than ryegrass from depth in soils and therefore to offer greater drought resistance (Durand et al. 2007; Ghesquiére et al. 2010). Novel deep rooting *L. perenne* × *F. glaucescens* and *L.perenne* × *F. mairei* amphiploid hybrids have been developed at IBERS and have demonstrated excellent agronomic traits with the potential for improved efficiency of ruminant nutrition (Humphreys et al. 2014b). An introgression‐breeding approach similar to that described above involving Caucasian clover has been used successfully in *Festulolium*. In this case, in two separate breeding programs, *F. glaucescens* and *F. arundinacea* genes were introduced onto different locations on chromosome 3 of *L. multiflorum* and subsequently also transferred into *L. perenne* (Humphreys and Thomas 1993; Humphreys et al. 2005, 2014a). In all instances, the water‐use‐efficiency was significantly enhanced compared to *Lolium*. Chromosome 3 was a good target for entry of novel allelic variants into *Lolium* for drought resistance as QTL for the trait have been detected throughout the chromosome in *Festuca,* but have never been reported there in *Lolium* species (Turner et al. 2008; Alm et al. 2011). Turner et al. (2008) reported a correlation between increased root growth and enhanced leaf extension under drought when certain *Festuca*‐derived genes were transferred onto chromosome 3 of *Lolium*. The forage production and quality of these different combinations of ryegrass and fescue is being studied (Humphreys et al. 2014b) in parallel with analysis of root ontogeny (MacLeod et al. 2013).

Selection for above‐ground biomass, in parallel with below‐ground biomass, is part of the IBERS forage breeding programme within the “Public Good Plant Breeding Group”. The “SUREROOT” project funded through the Biotechnology and Biological Research Council LINK programme includes an element of selection for root architectural traits, especially root depth and thickness. This research has highlighted the challenge facing plant breeders of developing appropriate methodologies to phenotype root traits to quantify the variation in root traits within breeding populations.

The following sections summarize the new technologies being used to quantify variation in root architecture between and within species and in response to specific management strategies.

### Phenotyping approaches

Phenotyping of root traits presents technical challenges, particularly in relation to the number of plants that must be screened in a plant breeding program. Typical approaches, such as those described by Chmelikova et al. (2015) for analysis of red clover roots, involve digging up a soil monolith beneath the plant and washing to extract the below‐ground organs of the plants from the soil. The root mass of the whole plant is then digitized using a scanner and the number and size of roots quantified. This often leads to underestimation of fine roots through breakage during washing, and the three dimensional spatial distribution is also lost (Mairhofer et al. 2013). Alternative nondestructive approaches have included the analysis of root systems in flowing solution culture (Abberton et al. 2000; Collins et al. 2003), but the relevance of these results to performance in the field is often questioned. A more recent approach has been the application of automated scanning technology to reconstruct a three‐dimensional data set. CT X‐ray tomography, for example, has been used to analyze root architecture of plants grown in soil (Mairhofer et al. 2013) and to analyze the effect of soil moisture on root architecture (Zappala et al. 2013).

**Figure 1:**
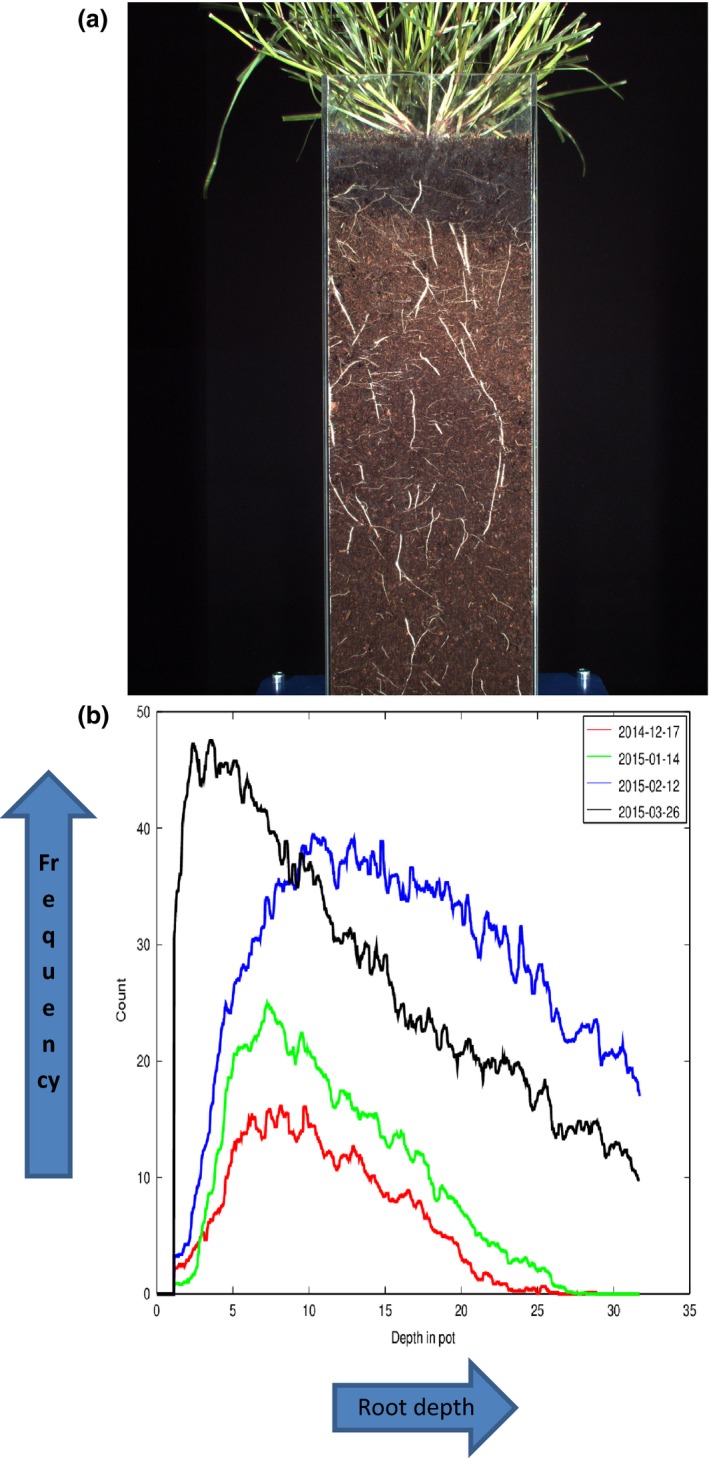
(a) Festulolium grass hybrid growing in potting compost within a 12 × 12 × 50 cm clear column for root analysis over consecutive months in the National Plant Phenomics Centre at IBERS and (b) Root ontogeny measures derived from comparisons in root density scores of a Festulolium grass hybrid taken over 4 consecutive months. Mean root density scores are calculated from 12 merged root images each representing consecutive 5 cm sections along a 50 cm root column. High vis camera images were captured and merged at the National Plant Phenomics Centre at IBERS.

Other phenotyping approaches are also available to study the root architecture of forage grasses and legumes and to quantify the impact of different root architecture on water flow and nutrient dynamics. For example, the NPPC at IBERS provides dynamic (nondestructive) developmental and physiological imaging of shoots and roots of plants automatically moved from controlled environments to imaging systems. This state‐of‐the art system is designed for automatic, high throughput, nondestructive phenotyping of a wide range of plant material. Near infrared (NIR) thermography provides multiple‐sided imaging of roots and soil, to detect root growth and soil water content profile changes for plants grown in root columns. In this system, root columns are transported on carriages identified by RFID tags allowing each plant to be imaged, and provided with precise watering and nutrition (or, if necessary, sprayed). Watering, nutrient, and weighing stations allow precise control on a per‐plant basis for controlled drought, nutrient, and water stress experiments. An example of an output of the detailed monitoring of monthly changes in root distribution and number for a *Festulolium* hybrid‐derivative is shown in Fig. 1.

### Plant testing systems

The varieties of grasses and legumes used in agricultural and amenity grassland systems within the EU must meet certain standards if they are to be marketed. A series of protocols that determine their DUS (Distinctiveness, Uniformity and Stability) and VCU (Value for Cultivation and Use) are used to identify the best varieties for use (Gilliland and Gensollen 2010). The DUS system is designed to provide protection for the intellectual property rights (IPR) residing in existing novel varieties and is controlled by legislation in all EU member states, conducted in compliance with international guidelines compiled by UPOV (The International Union for the Protection of New Varieties of Plants). Assessing the VCU of forage grasses and legumes is subject to national statutory testing within all EU member states. Such systems seek to ensure that new varieties reach a certain level of performance before seed of those varieties can be sold commercially. New plant varieties are therefore evaluated in agronomic trials with control varieties, and only those that reach a certain level of performance are added to the UK Common Catalogue and can be sold. However, there are no internationally agreed guidelines within the EU and no harmonized testing protocols between official testing authorities. The testing systems used differ between species and countries, and for agricultural species they focus specifically on the evaluation of agronomic traits such as dry matter yield and disease resistance, with relatively limited analysis of forage quality characteristics (Gilliland and Gensollen 2010). Considerable emphasis is now being placed on the measurement of nutritional parameters that can quantify “forage value” to farmers in terms of direct ruminant benefit. Despite these developments, the current systems are not designed to quantify the merits of improved forage varieties in terms of ecosystem services. Development of appropriate tests that analyze the value of forage varieties in terms of delivery of such services is therefore unlikely and probably commercially unsustainable. Testing systems need to be robust with relatively simple protocols, necessitating decisions on which “ecosystem services” are to be targeted, followed by the development of appropriate protocols for measuring the impact of plant traits on these services and ranking them in comparison with agronomic and forage quality traits.

## Conclusions

This paper has concentrated on the capacity of grassland ecosystems to deliver environmental benefit, and the challenge of balancing the delivery of these benefits whilst maintaining the production of high‐quality forage for livestock production. It has focused on the potential for the genetic improvement of specific root traits within the forage species that are currently being used in UK grassland agriculture. Within the scope of this review, it was not feasible to consider the potential opportunities from inclusion of alternative forage species or to consider the impact that different grassland management systems may have on the ability of grassland ecosystems to deliver these environmental services, but these should be considered important factors when assessing the potential of grassland systems.

## Conflict of Interest

The authors declare that they have no conflict of interest.
